# Lumbar Intervertebral Motion in Healthy Male Participants: Protocol for a Motion Analysis During Flexion and Extension Cinematographic Recordings

**DOI:** 10.2196/14741

**Published:** 2020-03-03

**Authors:** Inge J M H Caelers, Toon F M Boselie, Kim Rijkers, Wouter L W Van Hemert, Rob A De Bie, Henk Van Santbrink

**Affiliations:** 1 Care and Public Health Research Institute for Public Health and Primary Care Maastricht University Maastricht Netherlands; 2 Department of Neurosurgery Zuyderland Medical Centre Heerlen Netherlands; 3 Department of Neurosurgery Maastricht University Medical Centre Maastricht Netherlands; 4 Department of Orthopaedic Surgery Zuyderland Medical Centre Heerlen Netherlands; 5 Department of Epidemiology Maastricht University Maastricht Netherlands

**Keywords:** fundamental research, lumbar spine, cinematographic recordings, motion pattern, flexion, extension, rotation, translation

## Abstract

**Background:**

Physiological motion of the lumbar spine is a subject of interest for musculoskeletal health care professionals, as abnormal motion is believed to be related to lumbar conditions and complaints. Many researchers have described ranges of motion for the lumbar spine, but only a few have mentioned specific motion patterns of each individual segment during flexion and extension. These motion patterns mostly comprise the sequence of segmental initiation in sagittal rotation. However, an adequate definition of physiological motion of the lumbar spine is still lacking. The reason for this is the reporting of different ranges of motion and sequences of segmental initiation in previous studies. Furthermore, due to insufficient fields of view, none of these papers have reported on maximum flexion and extension motion patterns of L1 to S1. In the lower cervical spine, a consistent pattern of segmental contributions was recently described. In order to understand physiological motion of the lumbar spine, it is necessary to systematically study motion patterns, including the sequence of segmental contribution, of vertebrae L1 to S1 in healthy individuals during maximum flexion and extension.

**Objective:**

This study aims to define the lumbar spines’ physiological motion pattern of vertebrae L1, L2, L3, L4, L5, and S1 by determining the sequence of segmental contribution and the sequence of segmental initiation of motion in sagittal rotation of each vertebra during maximum flexion and extension. The secondary endpoint will be exploring the possibility of analyzing the intervertebral horizontal and vertical translation of each vertebra during maximum flexion and extension.

**Methods:**

Cinematographic recordings will be performed on 11 healthy male participants, aged 18-25 years, without a history of spine problems. Cinematographic flexion and extension recordings will be made at two time points with a minimum 2-week interval in between.

**Results:**

The study has been approved by the local institutional medical ethical committee (Medical Research Ethics Committee of Zuyderland and Zuyd University of Applied Sciences) on September 24, 2018. Inclusion of participants will be completed in 2020.

**Conclusions:**

If successful, these physiological motion patterns can be compared with motion patterns of patients with lumbar conditions before or after surgery. Ultimately, researchers may be able to determine differences in biomechanics that can potentially be linked to physical complaints like low back pain.

**Trial Registration:**

ClinicalTrials.gov NCT03737227; https://clinicaltrials.gov/ct2/show/NCT03737227

**International Registered Report Identifier (IRRID):**

DERR1-10.2196/14741

## Introduction

Physiological motion of the lumbar spine is a subject of interest for musculoskeletal health care professionals. Although *physiological motion* is used in many instances, a proper definition is still lacking. More knowledge about physiological motion is essential to recognize abnormal motion caused by specific diseases, complaints, or medical interventions.

In 1929, Virchow et al [1] were the first to use sagittal radiographs to analyze normal range of flexion and extension of the cervical spine. In 1931, Dittmar et al [2] were the first to perform this type of research for the lumbar spine. Troke et al [3] developed a database of healthy individuals, aged 16-90 years, with ranges of motion from Th12 to S1. After these studies, more motion and range of motion research using radiographs followed, and later on, computed tomography– or magnetic resonance imaging–based 3D images were used[4-6]. However, ranges of motion have a high intra- and interindividual variability [3,7]. For this reason, recent studies have described the motion of individual segments and the sequence of segmental initiation of motion in flexion and extension of the lumbar spine. However, these studies still reported different motion sequences. This lack of a consistent sequence hampers the definition of *physiological motion* of the lumbar spine.

Boselie et al [8] have recently described a rather consistent sequence of segmental contribution in sagittal rotation during flexion and extension in the lower cervical spine. This research was used to create a definition of physiological motion. To our knowledge, research on the sequence of segmental contribution has not been carried out for the lumbar spine.

The aim of this study is to analyze the lumbar spine, regarding the sequence of segmental contribution and the sequence of segmental initiation of motion of L1 to S1 in sagittal rotation during flexion and extension in individual participants. Additionally, the researchers will explore the possibility of analyzing intervertebral horizontal and vertical translation to enable determination of the sequence of segmental contribution and the sequence of segmental initiation of motion of L1 to S1.

Sagittal cinematographic recordings will be conducted during lumbar flexion and extension in asymptomatic male participants to determine the sequences. If a consistent pattern of segmental contributions is found in asymptomatic participants, this pattern can be used to investigate potential abnormal motion in conditions in the future. Differences in biomechanics may result in physical complaints.

## Methods

### Participants, Recruitment, and Study Setting

Participants will be recruited at two hospitals and two universities of applied sciences by using information posters. Potential participants can send an email to author IC. IC will evaluate eligibility and inform participants about the study verbally and in writing. They will be contacted again at least 1 week later to inquire if they are willing to participate.

This study will include men aged 18-25 years with a Body-mass Index<25 kg/m^2^, no medical history of spine problems and who are able to perform maximum lumbar flexion and extension without complaints. No medical history of spine problems is defined as no visits to a doctor for spine complaints, no former spine surgery, Oswestry Disability Index and Visual Analogue Scale scores of zero for back pain, and Kellgrens’ classification of 0-1 in levels L4L5 and L5S1 on cinematographic recordings evaluated by two neurosurgeons or orthopedic surgeons (authors TB, HvS, WvH, or KR) [9-11]. Potential participants are excluded if x-rays were taken of the abdomen, pelvis, hip, lumbar, or sacral spine in the previous year or in cases of active spinal infection, immature bone, lumbar tumor, previous lumbar radiotherapy, congenital lumbar spine abnormality, or planned pregnancy of the participants’ partner in the coming year. There are two reasons for these strict inclusion and exclusion criteria. First, only asymptomatic healthy participants are included, because they reflect normal motion. Second, the criteria minimize radiation exposure of participants during the study. Informed consent will be acquired from all participants.

### Sample Size

Sample size calculation is based on previous studies that used the same method of cinematographic recordings to analyze spine motion. Boselie et al [12], Kanayama et al [13], and Harada et al [14] used 8-10 participants to perform adequate analysis and draw solid conclusions. To minimize radiation exposure in healthy participants, we will not include more participants than necessary for analysis. This results in a study population of 11 participants, assuming an expected maximum loss to follow-up of 10%. Flexion and extension cinematographic recordings are acquired twice for each participant with an interval of 2 weeks in order to determine reproducibility and consistency of sequence of motion between two time points (T1 and T2) [8,15].

Participants can cease study participation at any time for any reason without consequence. If participants leave the study before the second recording, only the first recording will be included and analyzed. Researchers can only withdraw participants that do not respond to calls before the first cinematographic recording or if abnormalities of the lumbar spine are observed during the first cinematographic recording.

### Study Procedures

In order to acquire cinematographic recordings of flexion and extension of the lumbar spine, participants will be seated in a chair designed to set the pelvis in a fixed position. They will be instructed to perform maximum extension, followed by maximum flexion, and then return to maximum extension in 14 seconds using a metronome. Cinematographic recordings will be made from a lateral perspective to obtain sagittal images. Participants perform these recordings twice with a 2-week interval.

### Radiological Outcome Measures

The outcome of this study will define (1) the sequence of segmental contribution in rotation and, if possible, translation during flexion and extension and (2) the sequence of segmental initiation of motion in rotation and, if possible, translation during flexion and extension

### Radiological Data, Radiological Acquisition, and Radiation Dose Calculation

Cinematographic recordings are made using the Philips Allura Xper FD20 x-ray system, capturing frames of 1024x1024 pixels at 7.5 frames per second. Radiation dose per cinematographic recording, determined by radiation experts, will be around 0.21 mSv. The settings used for calculation are an exposed tissue factor of 0.54 mSv/Gy (based on International Commission on Radiological Protection 103), tube voltage of 75-90 kV, record duration of 20 seconds, filter of 0.9 mm copper + 1 mm aluminum, 7.5 frames per second, focus-detector distance of 48 cm, and a field of view of 520 cm^2^. Participants will perform cinematographic recordings twice, resulting in a total radiation dose of 0.42 mSv. This amount of radiation can be categorized in category IIa using the Neurocritical Care Society guidelines on risks of radiation dose (0.1-1.0 mSv) [16]. This category includes moderate risk that can be justified if there is a potential health benefit for future patients.

### Radiological Data Processing

The researchers have developed custom software that uses image recognition algorithms to track vertebrae during flexion and extension throughout these series of frames [17]. The software follows bony structures within user-defined template areas throughout all frames using a best-fit principle to match normalized gradient field images. To define these template areas, the user draws polygons around all vertebrae on the median frame of the recording [17]. After the software has completed tracking these structures, they can be manually evaluated, and corrections can be made if necessary. The rotational data between frames for each bony structure enables the user to calculate segmental ranges of motion and sagittal rotations within a motion segment through time. The sequence of various segmental contributions to movement of the entire lumbar spine can therefore be established.

Radiological data will be stored on CDs coded with participant number and recording number (T1 or T2). The CDs will be locked up in a secured room in the hospital and kept for 15 years after end of the study. Handling of personal data will comply with the guidelines of the Dutch Personal Data Protection Act.

### Interim Analysis

Interim analysis will be performed after cinematographic recordings of 2 participants, to determine if the images acquired with the Allura Xper are appropriate to perform computer software analysis. If segment L1 does not remain in the field of view, analyses will start downwards from segment L2.

### Radiological and Statistical Analysis

Computer analysis will be performed by IC for all cinematographic recordings. Graphs will be made for flexion and extension. Segmental rotation (cumulative and between each pair of successive frames) of each individual segment L1 to S1 will be plotted against the cumulative rotation in segments L1 to S1 together, to describe the sequence of segmental contribution and sequence of segmental initiation of motion. These graphs will be made and analyzed for each individual participant to identify specific patterns in the sequence of segmental contributions. If possible, a sequence definition will be described for segmental contribution and initiation of motion in flexion and extension of the lumbar spine. Analysis will first be performed for T1 and then tested against T2 using the kappa coefficient to determine intraindividual variability.

Ten recordings will also be evaluated by second researcher TB to determine reproducibility when using two-way mixed intraclass correlation coefficient testing. If the intraclass correlation coefficient for intervertebral horizontal and vertical translation is higher than 0.60, the sequence of segmental contribution and sequence of segmental initiation of motion of intervertebral horizontal and vertical translation will be determined as well. If the intraclass correlation coefficient is less than 0.60, intervertebral horizontal and vertical translation will not be determined because reliability of results will be insufficient.

### Data Monitoring, Safety Reporting, and Publication

Monitoring will be performed by independent, trained, and qualified monitors according to the good clinical practice guidelines. Monitoring will be performed three times: one site initiation visit, one interim monitoring visit, and one close out visit. Adverse events will be collected by questionnaires during T1 and T2. Adverse events will be followed until they have abated or until a stable situation has been reached. Serious adverse events will be reported through the Web portal ToetsingOnline to the accredited Medical Ethical Review Committee (METC) that approved the protocol. The researcher has a liability insurance that provides coverage for damage to research participants because of injury caused by the study.

Possible amendments of the study protocol have to be approved by the accredited METC.

Results of the study will preferably be published in open access, peer-reviewed journals. Data of participants will be anonymous and untraceable to any individual. Authors will not be able to veto whether to publish data.

## Results

The study has been approved by the local institutional medical ethical committee (Medical Research Ethics Committee of Zuyderland and Zuyd University of Applied Sciences) on September 24, 2018. Furthermore, it was registered on ClinicalTrials.gov (NCT03737227) on November 9, 2018. Inclusion of participants will be completed in January 2020, followed by analysis of cinematographic recordings.

## Discussion

The aim of this study is to describe the sequence of segmental contribution and the sequence of segmental initiation of motion in sagittal rotation and translation during maximum flexion and extension of the lumbar spine in asymptomatic male participants, with the intention of finding a consistent and reproducible motion pattern. In the future, the researchers will aim to use this physiological motion pattern to compare with potentially abnormal motion patterns in patients with lumbar spinal conditions or in patients after spine surgery to determine if differences in biomechanics are present, which may result in physical complaints.

Previous studies have used different imaging techniques to describe the range of motion and initiation of motion of individual segments during flexion and extension of the lumbar spine. Extension seems to correlate to a smaller translation and range of motion compared to flexion, where most studies describe L5S1 as least mobile. However, those studies reported different sequences and have not described motion patterns of maximum flexion and extension for L1 to S1 due to insufficient fields of view [8-13,18-26]. Furthermore, all studies described cumulative rotation of each individual segment at specific time points or at specific lumbar ranges of motion, which can result in missing drastic changes in intervertebral rotation between successive frames.

This study has several strengths. First, this study will use strict inclusion criteria to select only young adult male participants without low back complaints. Females are excluded to protect their ovaries from direct radiation exposure. However, Dvorak et al [19] and Wong et al [26] have shown that there is no statistically significant difference between sexes in motion of the lumbar spine. Comparable results were described by Boselie et al [8] for the cervical spine. Second, the researchers want to describe a more thorough motion pattern of the total lumbar spine, including information about all segments from L1 to S1 during maximum flexion and extension; sequences of segmental contribution (except the already described sequence of segmental initiation of motion); and information on rotation and, if possible, translation in the sagittal plane. This study will describe the definition of the physiological motion pattern based on information in only one plane. However, based on the study of Boselie et al [8], the researchers can conclude that the description of motion patterns in only one plane is a consistent parameter and can be used to differentiate between symptomatic and asymptomatic patients.

This study provides a clearer conclusion of physiological motion patterns of the lumbar spine. This will eventually be compared to motion patterns of patients with lumbar conditions or who have received lumbar surgery.

## Abbreviations

METC: Medical Ethical Review Committee
T: time point
